# Problematic Social Media Use and Its Relationship with Breastfeeding Behaviors and Anxiety in Social Media-Native Mothers: A Mixed-Methods Study

**DOI:** 10.3390/healthcare13172216

**Published:** 2025-09-04

**Authors:** Hülya Tosun, Hava Özkan

**Affiliations:** 1 Midwifery Department, Health Science Faculty, Kütahya Health Science University, Kütahya 43020, Türkiye; 2 Midwifery Department, Health Science Faculty, Atatürk University, Erzurum 25030, Türkiye; havaoran@atauni.edu.tr

**Keywords:** problematic social media use, anxiety, breastfeeding attitudes, infant feeding intention, perinatal education, social media-native mothers

## Abstract

**Background/Objectives:** Social Media Use has become an integral part of contemporary motherhood, with potential effects on maternal mental health and breastfeeding behaviors. This study aimed to examine the relationship between problematic social media use, anxiety levels, infant feeding attitudes, and feeding intentions among social media-native mothers. **Methods:** A mixed-methods design was used with 222 mothers. Quantitative data were collected using the Trait Anxiety Inventory (STAI-T), Social Media Disorders Scale (SMDS-9), Iowa Infant Feeding Attitude Scale (IOWA), and Newborn Feeding Intention Scale (IFI). Qualitative data were obtained through semi-structured interviews. Quantitative analyses explored associations between problematic social media use, anxiety, and feeding-related outcomes, while qualitative data were analyzed thematically. **Results:** SMDS-9 scores were generally low; only 2.7% of participants reported low feeding intention. No significant association was found between problematic social media use and breastfeeding intention or attitudes. Mothers with strong breastfeeding intentions demonstrated significantly more positive attitudes toward nursing than those with lower intentions. Higher problematic social media use was observed among high school graduates and those from high-income groups. Qualitative analysis identified two main themes of Negative Impacts and Positive Impacts, as well as five sub-themes of comparison and inadequacy, information overload, breastfeeding mistakes, anxiety, and social support. **Conclusions:** Problematic social media use did not directly affect breastfeeding intentions or attitudes; however, high school-educated and high-income mothers were more likely to report higher usage. Incorporating social media literacy into prenatal education and adapting tools for assessing postpartum anxiety to include indicators for problematic social media use, particularly for these at-risk groups, may support early detection, reduce stress, and promote positive breastfeeding practices.

## 1. Introduction

The emergence of social media-native mothers, also known as digital natives, born after 1989 and maturing entirely within social media environments, has brought new dynamics to the motherhood experience [1,2]. For these mothers, social media is not merely a tool for information but also a medium for social contact, emotional support, and self-representation [3].

The increasing integration of social media platforms into maternal life has positioned their impact as a key focus in contemporary research [4]. The World Health Organization characterizes the postpartum period as one of the most susceptible but least supported stages in a woman’s life [5]. The increased psychological susceptibility during this time amplifies the influence of social media interaction on mental health and parenting practices [6].

While social media can offer informational and emotional benefits, problematic social media use (PSMU) has emerged as a significant public health concern for social media-native mothers [7]. On the other hand, problematic social media use is defined by excessive, uncontrolled, and compulsive interaction with social media platforms, adversely impacting individuals’ social interactions, daily functioning, and mental health [8,9,10]. In our study, problematic social media use refers to excessive, compulsive, and uncontrolled engagement with social networking platforms that results in functional impairment in daily life, emotional distress, or both [11,12]. Unlike normative use, characterized by purposeful and time-limited engagement, PSMU is marked by persistent use despite awareness of its negative consequences, interference with offline responsibilities, and an inability to reduce use voluntarily. This distinction is theoretically grounded in the *Self-Regulation Model* [13] which posits that problematic use arises when self-control mechanisms fail, and in the *Compensatory Internet Use Theory* [14] which explains how individuals may turn to online environments to cope with stress or unmet psychological needs, thereby reinforcing maladaptive usage patterns. Empirically, the PSMU was operationalized in this study using the Social Media Disorders Scale (SMDS-9) [15]. By applying this threshold, the present study distinguishes social media-native mothers with potentially harmful usage patterns from those engaging in frequent but non-problematic use.

PSMU can manifest during pregnancy and persist throughout the postpartum period, resulting in negative consequences such as sadness, stress, anxiety, sleep difficulties, and diminished work performance [16,17,18]. Its use among social media-native mothers can be understood through multiple, complementary theories. *Social Cognitive Theory* suggests that individuals learn and replicate behaviors by observing others, particularly when these behaviors are rewarded or idealized in online environments [19]. This theoretical framework elucidates how mothers may internalize parenting norms and feeding practices modeled by social media influencers or peers. The Inadequate Self-Regulation framework suggests that problematic media use arises when individuals are unable to control impulses and seek immediate gratification. In the postpartum context, mothers experiencing heightened stress may over-engage with social media as a source of emotional comfort, which can inadvertently detract from their attention to infant care [20]. Complementarily, Self-Discrepancy Theory posits that psychological discomfort emerges when one’s perceived self-image diverges from idealized standards. Exposure to *perfect motherhood* portrayals online can intensify feelings of inadequacy and anxiety among mothers, particularly in relation to breastfeeding and caregiving responsibilities [21].

Similarly, *Social Comparison Theory* [22] predicts that frequent upward comparisons, such as comparing oneself to idealized mothers on social media, can intensify postpartum anxiety and reduce maternal self-confidence [18]. *Media Dependency Theory* emphasizes how reliance on digital platforms for information, reassurance, and social approval can reinforce dependency patterns, shaping maternal attitudes and feeding intentions [23]. Complementing this, *Diffusion of Innovations Theory* explains how new practices and behaviors, such as specific feeding approaches promoted online, spread through social networks and are adopted or rejected by mothers [24].

Rather than operating in isolation, these theories collectively suggest a process in which postpartum mothers engage with social media to meet informational, emotional, and social needs. However, over time, idealized portrayals and constant comparisons can erode maternal self-efficacy, increase anxiety, and influence breastfeeding behaviors. The important phenomenon, “super motherhood”, which has found its place more dominantly in social media in recent times, is driving mothers into anxiety. Super motherhood refers to the cultural idealization of motherhood as a flawless, all-capable, self-sacrificing, and emotionally composed role, rooted in the ideology of intensive mothering, which positions the mother as the central caregiver responsible for meeting every need of the child to the highest possible standard [25]. In contemporary contexts, particularly on social media, this ideal is reinforced through curated portrayals of domestic competence, physical appearance, and child development milestones. While such representations may inspire some mothers, they often create unrealistic expectations, leading to feelings of inadequacy, guilt, burnout, and increased anxiety, especially among first-time mothers navigating new maternal roles [26,27]. Exposure to these ideals can also result in unhelpful social comparisons and fear of criticism, further heightening stress levels [27]. A study conducted by Aker et al. [28] showed that 59% of working mothers experienced feelings of guilt and inadequacy stemming from social media. This situation encourages them to adopt a more permissive parenting approach and to overlook mistakes in childcare in an effort to compensate for their guilt.

In addition to psychological effects, social media usage influences physiological and behavioral systems. Engagement in tasks necessitating concentration and duration, such as breastfeeding, while actively using social media may diminish eye contact and physical touch with the newborn as well as hinder bonding [28]. Furthermore, social media utilization has been demonstrated to directly influence breastfeeding length, parental breastfeeding self-efficacy, and feeding motivation [29,30]. Excessive social media usage considerably diminishes a mother’s urge to breastfeed [29,31]. Another negative effect of social media on mothers is the constant exposure to idealized representations of parenting and body images. Such exposure distorts mothers’ judgments about their own body image and reduces their self-efficacy [32,33]. Admiration or envy toward idealized mothers increases maternal anxiety. Consequently, breastfeeding and appropriate infant feeding practices are adversely affected [33,34]. Feelings of inadequacy and stress caused by excessive social media use have been shown to lead to inappropriate transitions to complementary feeding and to decrease mothers’ breastfeeding confidence [9]. In addition, exposure to aggressive formula marketing on social media has been linked to a decline in breastfeeding rates [35]. Furthermore, research has shown that high levels of social media use after childbirth are associated with postpartum depression. These findings highlight the importance of examining the relationship between problematic social media use and maternal mental health in terms of depression and other mental health problems [35,36].

The utilization of social media by contemporary mothers is closely related to their parenting practices, particularly breastfeeding motivation and postpartum anxiety. According to *Media Dependency Theory* [23] and *Diffusion of Innovations Theory* [24], the robust relationship established with social media fulfills the need for information, security, and social affiliation. Nonetheless, the continual satisfaction of these requirements in digital environments may cultivate digital dependency and undermine conventional parenting practices.

This study aims to evaluate the relationships between problematic social media use, trait anxiety, infant feeding attitudes, and feeding intentions among social media-native mothers. A clearer understanding of these associations may guide the development of targeted interventions to foster healthy social media use and enhance maternal well-being during the postpartum period.

## 2. Method

An Explanatory Sequential Mixed Methods Design approach was employed by prioritizing the collection and analysis of quantitative data. This design is particularly appropriate when numerical findings alone are insufficient or when unexpected or complex results require further exploration. By combining statistical trends with participants’ perspectives, the explanatory sequential approach allows researchers to answer both the “what” and the “why” of the research question, resulting in a more comprehensive understanding of the phenomenon under study [37]. In this study, the Explanatory Sequential Mixed Methods Design was implemented through the following steps: (1) quantitative data collection, (2) quantitative analysis, (3) identification of key results, (4) qualitative data collection, (5) qualitative analysis, and (6) integration and interpretation.

The study was conducted between 20 May 2022 and 20 August 2022 at the Obstetrics and Pediatrics Outpatient Clinics of Kutahya Health Science University, Kutahya Evliya Çelebi Training and Research Hospital, Türkiye. It purposefully selected a single, urban, hospital-based postpartum clinic in Türkiye because it (i) serves a large catchment area with socio-demographic heterogeneity, (ii) delivers standardized perinatal and lactation counseling under national *Baby-Friendly* protocols [38], and (iii) had existing clinical workflows and data-protection infrastructure that enabled timely ethical approval, recruitment, and mixed-methods data collection in the immediate postpartum period. A single-center design also reduced site-level heterogeneity in breastfeeding counseling, discharge instructions, and digital education practices, which is particularly important when examining subtle associations between social media exposure and psychosocial outcomes.

### 2.1. Participants

The study cohort comprised mothers aged 20–34 years in the postpartum period between 2 weeks and 6 months. Eligibility criteria included having a healthy infant, using Facebook and Instagram, being fluent in Turkish, and having no communication impairments.

For the quantitative phase, a large sample was recruited using probability-based sampling methods to enhance representativeness, and the sample size was determined through a priori power analysis G*Power 3.1 software [39]. At a significance level of 0.01, an effect size of 0.10, and a desired statistical power greater than 95%, a minimum of 228 participants was calculated. A total of 222 mothers completed the study, because some left parts of the forms blank (*n* = 4) and two people withdrew their consent to data use during the study.

Following the quantitative analysis, key results and subgroup characteristics guided the design of the qualitative phase. In this phase, a smaller sample was selected through purposeful sampling strategies, particularly criterion sampling and maximum variation sampling, to capture diverse perspectives. The final sample size for the qualitative phase was determined based on data saturation. Qualitative interviews were completed with 17 mothers. The qualitative sample size (*n* = 17) has been deemed sufficient in accordance with Creswell’s (2007) recommendations, as it enabled thematic saturation and in-depth exploration within a relatively homogeneous group of participants [37]. Consequently, the study’s overall validity was enhanced by the integration of generalizable quantitative findings with rich, in-depth qualitative insights, which was facilitated by this combined strategy.

All interviews were conducted face-to-face in a suitable hospital meeting room. A code name was assigned to each participant to ensure confidentiality and maintain the reliability of individual data.

In this study, reflexivity and positionality were carefully ensured. The first author, a female midwifery academic with clinical and research experience in maternal health, conducted all interviews. Acknowledging that the researcher’s professional background and personal perspectives could influence the data collection and interpretation, reflexive practices were applied throughout the study [40]. These included maintaining a reflexive journal to record assumptions, impressions, and methodological decisions, and engaging in regular research team meetings to discuss emerging findings and alternative interpretations. This process enhanced transparency, minimized potential bias, and ensured that the thematic analysis was grounded in participants’ narratives rather than the researcher’s preconceptions.

### 2.2. Hypothesis

**H1:**  *Higher problematic social media use is associated with increased maternal anxiety in the postpartum period.*

**H2:**  *Higher problematic social media use is associated with lower breastfeeding intentions.*

**H3:**  *Stronger breastfeeding intentions are associated with more positive breastfeeding attitudes.*

**H4:**  *Problematic social media use varies significantly by demographic factors.*

### 2.3. Ethical Considerations

The ethical approval was received from the Ethics Committee of the Faculty of Health Sciences, Atatürk University. All participants provided written consent and were assured of confidentiality. Since they volunteered to participate, they had the right to withdraw at any time without any pressure. The research procedures were conducted following the ethical standards outlined in the Declaration of Helsinki.

### 2.4. Data Collection Procedure

Data collection was performed during routine postpartum visits via the provided questionnaires and semi-structured interview questions. Participants completed all measures independently after being informed about the consent.

The *Personal Information Form* consists of an 8-item questionnaire intended to collect demographic and personal information. In addition, the *Infant Feeding Intentions (IFI)* Scale, by Nommsen Rivers and Dewey [41], has 5 items evaluated by a 5-point Likert scale to measure breastfeeding intentions. Total scores vary from 0 to 16, where higher values indicate greater motivation. Cronbach’s Alpha of this was 0.90. The Turkish adaptation by Er et al. [42] exhibited a Cronbach’s alpha of 0.86. The value of Cronbach’s alpha in this study is 0.83. *The Iowa Infant Feeding Attitude Scale (IIFAS)*, introduced by De La Mora and Russell [43] aims to evaluate women’s attitudes towards breastfeeding, forecast the infant feeding method, and estimate breastfeeding duration. The cumulative attitude score varies between 17 and 85. Cronbach’s alpha coefficient cited in three studies by De La Mora and Russell [43] was 0.86. Eksioğlu et al. [44] conducted the Turkish adaptation and validation of the scale, resulting in a Cronbach’s alpha coefficient of 0.71. The value of Cronbach’s alpha in this study is 0.66.

Participants’ levels of problematic social media use were assessed using the *Social Media Disorder Scale-9 (SMDS-9)*, the original form developed by Van den Eijnden et al., 2016 [15]. The 9-item scale has total scores ranging from 0 to 9. Higher scores indicate greater problematic use. Cronbach’s alpha coefficient for the scale varies from 0.72 to 0.86. The scale was adapted into Turkish by Sarıçam and Adam Karduz (2018) [45]. It employs an 8-point Likert-type frequency scale (0–7), yielding a total score between 0 and 63. Since no standardized cut-off has been established for this version, scale scores were treated as a continuous variable in the present study. Cronbach’s alpha was determined to be 0.75. The value of Cronbach’s alpha in this study is 0.79.

*The State-Trait Anxiety Inventory (STAI-T)* was introduced by Spielberger et al. [46] to assess levels of state and trait anxiety. It was adapted and validated by Öner and LeCompte [47] for Turkish from 1974 to 1977. The inventory consists of sub-scales of state anxiety and trait anxiety, each including 20 items. The study exclusively used the trait anxiety subscale, which evaluated individuals’ general emotional states using a 4-point Likert scale. Higher scores signify increased levels of anxiety, whilst diminished values imply reduced levels. The cumulative score varies from 20 to 80, where scores between 40 and 59 indicate moderate anxiety, 60 and 79 signify severe anxiety, and 80 represents extreme anxiety (panic level). The value of Cronbach’s alpha in this study is 0.82

*Semi-structured interview questions*; These procedures are consistent with best practices in qualitative research and adhere to the methodological standards recommended by Creswell et al. [40]. Content validity established for the semi-structured interview questions through a multistep process aligned with established qualitative research standards. Firstly, the interview questions were developed based on a comprehensive review of the existing literature and were informed by similar qualitative studies conducted by various researchers [18,22,48,49]. Secondly, the proposed questions were reviewed by four experts in maternal health and qualitative research. Their feedback was used to ensure the questions’ clarity, relevance, and alignment with the study’s objectives. Lastly, a pilot interview was conducted with two participants to assess the comprehensibility and appropriateness of the questions in practice. Minor revisions were made based on their feedback to enhance clarity and flow. The questions were as follows.

- In what ways has social media influenced your sense of self-efficacy or confidence in caring for and feeding your baby?
- Can you describe if and how using social media has increased your feelings of anxiety, especially regarding your role as a mother?
- Have you experienced any negative outcomes or consequences from problematic or excessive social media use during the postpartum period? Could you describe them?
- Can you share a time when social media content affected your breastfeeding practices, either positively or negatively?
- Do you believe that problematic social media use has influenced your emotional well-being or your relationship with your baby? How?
- What do you think are the biggest challenges or risks associated with using social media as a new mother, especially when it comes to feeding practices and emotional health?

The interviews, conducted face-to-face, lasted 45–60 min, were audio-recorded, and transcribed verbatim. The names used in the qualitative results of the discussion section are the code names given by the participants themselves.

### 2.5. Quantitative Data Analysis

The obtained data is analyzed using IBM SPSS Statistics version 22 (SPSS Inc., Chicago, IL, USA). Participants’ sociodemographic characteristics and scale scores of IFI, STAI-T, and IIFAS were examined. Before the main analyses, the normality of the scale total scores was assessed via skewness and kurtosis analysis. All the determined values were within the acceptable range of ±2, indicating normal distribution, so parametric tests were used for group comparisons. In addition, reliability analyses were conducted for each scale, confirming adequate internal consistency. Independent Samples *t*-tests were employed for comparisons involving two independent groups. Alternatively, One-Way Analysis of Variance (ANOVA) was conducted if there were more than two groups. In case of a significant difference, Scheffé’s test was used under the assumption of homogeneity of variance, while Tamhane’s T2 was performed when this assumption was violated. Descriptive statistics were presented as frequencies (n) and percentages (%) for categorical variables, while the means, standard deviations, skewness, kurtosis, minimum, and maximum values were provided for continuous variables. Correlational relationships between continuous variables were examined using Pearson correlation analysis, with a significance level set at *p* < 0.05.

### 2.6. Qualitative Data Analysis

The researchers transcribed the interviews verbatim and reviewed the transcripts multiple times to ensure an accurate understanding of the content and consistency in the inductive content analysis. Thematic analysis was conducted following the six-phase approach outlined by Braun and Clarke [50]: (1) familiarization with the data, (2) generation of initial codes, (3) searching for themes, (4) reviewing themes, (5) defining and naming themes, and (6) producing the report.

Each transcript was carefully examined throughout the analysis process to ensure an in-depth understanding of the data. The analysis was conducted by two researchers who independently read the transcripts and assigned topic codes to each section to reflect the subject under discussion. Subsequently, the individually developed codes were merged into a single coding system, and the process was repeated until consensus was reached. The generated codes led to the identification of sub-themes, which were then grouped into overarching themes.

The researchers observed that their own opinions and values did not influence the interpretations derived from the data. To ensure the reliability of the findings, strategies proposed by Braun and Clarke [50] were applied, including credibility (participant checking and peer debriefing), transferability (applicability to similar participants or contexts), and confirmability (posing methodological questions and maintaining data records).

## 3. Findings

Table 1 contains demographic information about the participants. The participants had primary school, middle school, high school, and university degrees-with 5.4%, 14.0%, 31.5%, and 49.1%, respectively. From an income perspective, most of them (59.5%) had an income equal to their expenses, while 17.1% of them had less. Currently, most of them (88.7%) are breastfeeding while the rest (11.3%) are not.

The IFI scores in Table 2 indicate that 2.7% of the participants have low newborn feeding intention. Cronbach’s alpha values of 0.838 were determined to be reliable.

The results in Table 3 indicate no significant relationship between the participants’ scale scores (*p* > 0.05).

The comparison of scale scores corresponding to the IFI levels is provided in Table 4. Those with low feeding intention also had low infant feeding behavior scale scores (*p* < 0.002).

When the scale scores of the participants were compared according to their demographic characteristics, no difference was determined in the number of children, current breastfeeding status, how many times a day the baby is breastfed, why they stopped breastfeeding (if not breastfeeding), and whether they switched to supplementary feeding. However, significant differences according to economic status and income levels can be seen in Table 5 and Table 6.

A significant difference is observed between the levels of education in terms of the total SMDS-9 scale score (*p* = 0.049 < 0.05). The results of the Post Hoc analysis showed that those with a high school education had a higher rate of social media use disorder (mean of 10.05) than those with a middle school education (mean of 6.54) (Table 5).

The results in Table 6 indicate that a significant difference was determined between the income levels in terms of the total SMDS-9 scale score (*p* = 0.044 < 0.05). Accordingly, the participants whose income was higher than their expenses had a higher social media use disorder (mean of 9.90) than those whose income was lower than their expenses (mean of 6.68).

In Table 7, the age and STAI-T scores (*p* < 0.05) indicated a significant positive correlation between the IIFAS total score and age (*r* = 0.147, *p* = 0.028 < 0.05). In addition, a significant negative correlation was determined between age and the SMDS-9 total score (*r* = −0.231, *p* < 0.001), indicating that the mother’s positive attitude towards breastfeeding will increase and social media use disorder will decrease as age increases. The results also indicate that there was a negative, significant, and very weak correlation between the duration of breastfeeding the previous baby and STAI-T (*r* = −0.242, *p* = 0.027 < 0.05). Accordingly, anxiety decreases as the duration of breastfeeding the previous baby increases. However, a significant, positive, and moderate relationship was determined between the baby’s starting time for complementary feeding and IIFAS (*r* = 0.508, *p* = 0.008 < 0.05). It showed that the time for babies to start complementary feeding also increases as the mothers’ positive attitudes towards breastfeeding increase.

The statistics between the demographic characteristics and the baby feeding intention scale scores are provided in Table 8. The participants’ breastfeeding status and IFI levels were determined to be statistically significant (*p* = 0.005 < 0.05). Accordingly, 77.2% of the participants who breastfeed their babies have a higher intention to feed their babies.

A significant relationship was also determined between the number of breastfeeding times and their intention to feed their babies (*p* = 0.017 < 0.05). Accordingly, 33.3% of those with a low intention to feed—breastfed 1–3, 1–7, and 7–10 times, while 72.1% of them had a medium intention to feed more than 10 times. In addition, those with a strong intention to feed (75.2%) breastfed more than 10 times (Table 8).

A significant relationship was determined between switching to supplementary food and the breastfeeding intention scale (*p* = 0.012 < 0.05). The results showed that 66.7% of those with weak breastfeeding intention switched to supplementary food, while 75.5% of those with medium intention intended to switch to supplementary food. On the other hand, 83.2% of those with a strong intention did not intend to switch to supplementary food (Table 8).

The comparison of IFI scores according to some of the infant characteristics and maternal age variables is provided in Table 9. A significant difference was determined in infant feeding intentions based on the duration of previous breastfeeding experience among participants (*p* = 0.010 < 0.05). Specifically, those with lower feeding intentions had breastfed their previous child for a shorter duration (a mean of 4 months) compared to those with stronger intentions (a mean of 18 months).

### Qualitative Findings

**Figure 1:**
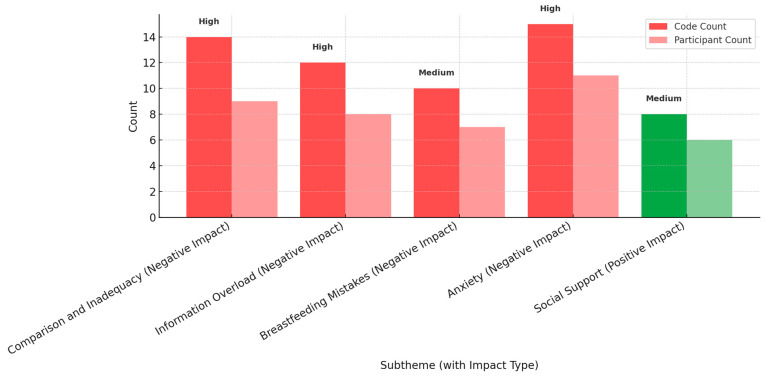
Code and participant counts across subthemes with importance levels. * Red and Light Red= Negative themes code count and, Participants count. ** Green and Light Green Positive theme code count and, Participants count.

The thematic analysis, conducted in line with Braun and Clarke’s (2006) framework, revealed both negative and positive aspects of social media use (Figure 1) [50]. Negative themes* were predominant, with Comparison and Inadequacy (code count = 14, participant count = 9, high), Information Overload (code count = 12, participant count = 8, high), Breastfeeding Mistakes (code count = 10, participant count = 7, high), and Anxiety (code count = 15, participant count = 11, high) emerging as the most prominent concerns.

In contrast, the only positive theme** identified was *Social Support* (code count = 8, participant count = 6, medium), which was mentioned less frequently. *Code count* represents the total number of times a theme was mentioned, while *participant count* reflects the number of distinct participants who discussed that theme.

## 4. Discussion

This study evaluated the relationship of problematic social media use among social media-native mothers on their postpartum breastfeeding intentions and anxiety levels, using a mixed-methods approach.

No statistically significant correlation was found between SMDS and breastfeeding intention. However, the mean SMDS scores in this sample were relatively low, with limited variability, which reduced the statistical power to detect possible associations. This limitation has been considered when interpreting the findings. The absence of significant relationships does not rule out the possibility that such associations may emerge in populations with higher or more variable levels of PSMU. Future research should explore these relationships in larger, more diverse samples, particularly those with higher SMDS-9 scores.

The existing literature provides inadequate empirical evidence demonstrating a negative association between inappropriate media use and breastfeeding outcomes. The majority of studies suggest that social media facilitates breastfeeding. Orchard and Nicholls [51] revealed that social media advertising indirectly enhanced the inclination to breastfeed. The study by Inoue [30] revealed that mothers could utilize their smartphones while concurrently supervising their infants, suggesting that smartphone usage did not diminish their attention to their newborns and breastfeeding. Researchers who warn to be cautious about this issue, for example, Srivastava et al. [48] contend that social media serves as a double-edged sword for breastfeeding, possessing the potential for both beneficial and detrimental effects.

When we look at the negative relationships between breastfeeding and social media, Mason et al. [52] stated that women’s engagement with social media while breast-feeding diminishes the quality of breastfeeding but does not directly influence breastfeeding trends. Morley and Owen’s (2019) study found that women with lower body image were less likely to initiate or intend to breastfeed, and those who initiated breastfeeding were less likely to breastfeed for a shorter duration [53].

In our study 80% of participants had completed at least a high school education, their social media literacy may have been strong, which may have increased their breastfeeding intentions. Nevertheless, social media literacy in Turkey varies substantially by age, education, and urban–rural residence, which may limit the generalizability of these findings [5,54,55]. Moreover, strong cultural norms, extensive family involvement, and supportive national health policies such as Prenatal Education Programs and the Baby-Friendly Hospital Initiative further promote breastfeeding, potentially enhancing mothers’ intentions [5,56]. This study found no significant link between PSMU and breastfeeding intentions among social media-native mothers, leading to the rejection of Hypothesis 2.

In this study, the majority of participants demonstrated a strong intention to breastfeed and a positive attitude toward breastfeeding. This finding confirms that breastfeeding intention is an important predictor of actual breastfeeding behavior [57]. In this study, postpartum women’s breastfeeding intention scale scores were consistent with those reported by Al Barwani et al. [58]. Evidence suggests that higher breastfeeding intention is associated with greater social and professional support for successful breastfeeding [58]. Additionally, mothers with stronger intentions have been found to exhibit more positive attitudes toward breastfeeding compared with those who have lower intentions [57]. This outcome aligns with the findings of other research examining the correlations among breastfeeding practices, certain demographic factors, and breastfeeding intentions [57,58]. Our findings confirm that breastfeeding intention is a strong predictor of actual breastfeeding behavior. This is consistent with previous studies showing that interventions that strengthen breastfeeding intention through education, skill development, and support networks can improve breastfeeding outcomes [59]. Results presented in this study that low breastfeeding intention was observed in 3% of mothers may parallel findings from a study in Australia [60] attributed to insufficient support, apprehension regarding stigma stemming from inadequate breastfeeding knowledge and skills, and residing in unfavorable environments.

This study revealed a notable correlation between mothers’ breastfeeding intention levels and their total scores on the IIFAS. Individuals demonstrating a more favorable disposition towards breastfeeding tend to possess elevated IIFAS scores. This finding aligns with numerous research studies undertaken at both national and international levels. A study by Cole et al. [61] revealed that the average IIFAS score of women intending to breastfeed was considerably higher than that of those not intending. Chekol et al. [62] similarly proved in their study with adolescent mothers that IIFAS scores effectively predict breastfeeding intentions and habits. Research in Türkiye has revealed a substantial correlation between mothers’ IIFAS scores and their attitudes towards nursing, indicating that those with elevated IIFAS scores are more likely to favor and persist in breastfeeding [63]. Hypothesis 3, social media-native mothers in the postpartum period exhibit strong intentions to breastfeed, is supported in this study.

In this study, no statistically significant association was found between postpartum anxiety and problematic social media use among social media-native mothers, although their mean postpartum anxiety scores were observed to be within the moderate range. This discovery is in stark contrast to certain findings in the existing literature. For instance, Lopes et al. [9] found that emotionally fraught interactions can contribute to elevated anxiety levels, despite the fact that the duration of social media use is not necessarily linearly related to anxiety. They also proposed that the impact of social media on anxiety may be indirect; idealized depictions of motherhood on these platforms can elicit feelings of inadequacy in certain mothers. In addition, social media can exacerbate self-comparison, a phenomenon that is associated with negative affect, diminished self-esteem, and jealousy, particularly in individuals with depressive symptoms [64]. *Social Comparison Theory* [22] supports these findings, while previous studies have indicated that time spent on platforms like Facebook is positively associated with depression [65]. During the perinatal period, body image concerns may further exacerbate mothers’ feelings of inadequacy [66].

In this study, social media-native mothers with a high school education were found to have higher levels of problematic social media use compared to those with only a middle school education. Interestingly, women with higher education degrees in the postpartum period tend to use social media more effectively for information on baby care and breastfeeding [18]. Similarly, prior research has shown that individuals with a high school education demonstrate higher scores in social media integration than those with other educational backgrounds [65]. Taken together, these findings suggest that mothers with a high school diploma may exhibit greater problematic social media use than those with lower education levels, potentially due to insufficient digital media literacy [67].

In this study another key finding was that participants whose income exceeded their expenses reported higher levels of problematic social media use than those whose expenses exceeded their income. This result may be explained by higher socioeconomic status, where individuals have more frequent access to digital platforms [68]. However, contrasting evidence from a Norwegian study suggests that a lower socio-economic situation (SES) increases the likelihood of negative social media experiences by 1.25 times. Individuals from low and middle SES groups were more likely to report exclusion and negative comments on social media compared to high SES individuals [69].

In this study, in terms of age, no significant relationship was determined between maternal age and postpartum anxiety. However, a positive association was observed between maternal age and breastfeeding intention. The findings are consistent with those reported by Bień et al. [70]. It can be suggested that increased maternal age may be associated with cognitive and experiential factors that positively influence feeding intentions. Greater maternal experience and improved access to health information over time may enhance awareness and commitment toward breastfeeding.

Furthermore, the results showed that problematic social media use scores tend to decrease with increasing age in this study. This suggests that age may play a regulatory role in social media behavior. Previous studies have shown that older individuals tend to engage with digital social media more selectively and purposefully, with increased cognitive awareness and life experience leading to more controlled use [71]. Similarly, Santini et al. [72] reported that younger individuals spend more time on social media and exhibit higher levels of digital dependence. Thus, age can be considered a moderating factor in digital media behavior, especially during life stages such as motherhood that require increased responsibility and self-regulation.

A significant negative relationship was also observed between the duration of previous breastfeeding and postpartum stress, indicating that maternal experience reduces postpartum stress in this study. These results are consistent with studies suggesting that increased maternal experience contributes to more confident and intentional breastfeeding behavior [73,74].

In this study another important finding was the association between the timing of complementary feeding and breastfeeding intentions. Mothers with more positive attitudes toward breastfeeding had a tendency for longer durations and introduced supplementary foods. These findings are parallel with Cox et al. [75], which suggests generational differences may not play a significant role in this domain. It also highlights the universality of maternal behavior and intention across different cohorts.

Additionally, a significant association was determined between social media-native mothers’ current breastfeeding status and their scores on the IIFAS in this study. Previous findings suggest that mothers with IIFAS scores above 65 are nearly twice as likely to exclusively breastfeed for the first six months and continue breastfeeding to 12 months at any intensity [75].

In this study, based on the evaluations of demographic data and scale scores, including maternal characteristics and infant-related variables, it was observed that these findings partially supported the H2 hypothesis. It is plausible that the measurement of multiple indicators has contributed to this outcome.

The thematic analysis, following Braun and Clarke’s (2006) [50] framework, revealed that mothers’ experiences with social media during the postpartum period were complex, encompassing both detrimental and beneficial aspects. While negative themes such as Comparison and Inadequacy, Information Overload, Infant Feeding Mistakes, and Anxiety were dominant, several participants also described positive or empowering experiences that improved their breastfeeding skills, confidence, and sense of social support [18,76]. Similarly to previous studies [17,77,78] the participants reported experiencing heightened anxiety due to social comparisons and exposure to conflicting information.

When examining the comparison and inadequacy subtheme, most participants indicated that social media increased their anxiety regarding their maternal roles. One mother expressed:

*“When I see on Instagram that other mothers take perfect care of their babies and also manage the house beautifully, I feel inadequate, and my stress rises. No matter how well I do, it still feels like I’m falling short.”*

*(Yasemin, age 24)*

These findings are consistent with Social Comparison Theory and align with prior research indicating that upward social comparisons are associated with reduced self-efficacy and increased psychological distress [79]. These relationships were especially evident among younger mothers, which may be attributed to their limited maternal experience and lower confidence in parenting abilities [35,79,80,81].

Infant feeding mistakes was another recurring issue, with some participants acting on unverified or conflicting advice encountered online. For example:

Another recurring issue was infant feeding mistakes, as some participants were acting on unverified or conflicting advice they encountered online. For example:

*“Seeing that others on social media introduced complementary foods to their babies at an early age influenced me… I thought this might be a convenience, and my sleep would not be interrupted. I introduced complementary foods at the fourth month, and my baby experienced severe gas problems. Nothing changed for me, I was sleepless again and my baby’s stomach aches made me feel guilty”*

*(Halime, 31 years)*

This finding corroborates prior evidence indicating that misinformation in online environments can exert a direct influence on maternal decision-making processes [82]. Within our sample, this subtheme frequently intersected with instances of infant feeding mistakes, suggesting that insufficient critical appraisal of online content may contribute to suboptimal feeding practices.

Anxiety was also a recurring emotional response. Some social media-native mothers initially sought guidance from social media but over time felt their confidence erode:

*“I’m tired of criticizing myself… Because influencer moms are always perfect. Our conditions are not equal, I know they exaggerated, but still, my self-confidence decreases.”*

*(Pelin, 34 years)*

Such experiences mirror Chee et al.’s (2021) findings that certain influencers exacerbate maternal self-criticism [80]. Additionally, problematic engagement patterns such as staying up late scrolling, neglecting self-care, or “phubbing” (ignoring real-life interactions) emerged in the accounts of several participants, reinforcing prior research linking excessive social media use with postpartum mental health challenges [81]. Two other social media-native mothers mentioned issues related to nutrition and fatigue caused by social media use:

*“I stay up very late, and the next day I feel tired and less tolerant toward my baby. Sometimes I let the baby cry too much, I cannot get up, and then I feel guilty.”*

*(Aylin, age 30)*

*“…when I spend time browsing the internet, I can’t eat properly, and it feels like my milk supply decreases.”*

*(Yeliz, age 25)*

This qualitative finding aligns with the results reported by Samra and Dryer [81], who found that problematic social media use was associated with higher levels of depression, pregnancy-related anxiety, and disordered eating attitudes.

Mothers who reported neglecting self-care expressed sentiments such as:

“I found myself constantly staring at my phone screen instead of playing with my baby. I would even cut my shower short just to get back to the screen. I felt terribly guilty…” (Şenay, age 21). These statements indicate that social media can particularly harm social media-native mothers, increasing postpartum anxiety levels and weakening mothers’ perceptions of self-efficacy [3,18].

When it comes to Information Overload, other participants shared the following:

*“I told a blogger that I felt my milk supply was low, and she told me it was because my breasts were small. How frustrating! She kept pushing me to eat more and more.”*

*(Ayşe, age 28)*

*“The marketed formula and milk-boosting products appeal to me. Sometimes I want to switch to formula so that the baby feels full and sleeps longer.”*

*(Asiye, age 23)*

These excerpts highlight how social media shapes maternal nutritional decisions by exposing mothers to commercial content and misinformation [82]. The narratives illustrate how persuasive marketing messages and unverified recommendations may distort feeding choices, thereby reinforcing a cycle of information overload, heightened anxiety, and ultimately suboptimal feeding practices [36,77,82].

Some social media-native mothers shared that social media improved their breastfeeding practices and infant care. They are feeling social support about it:

*“Thanks to the videos of a breastfeeding consultant on Instagram, I corrected my baby’s latch. It used to hurt a lot, and I was thinking about quitting.”*

*(Buse, age 27)*

*“From a baby care website, I learned how to take care of myself as well. That was important and made me feel so much more at ease.”*

*(Ceyda, age 22)*

This finding suggests that when used appropriately, social media can provide emotional support to mothers and positively influence breastfeeding practices [83]. The supportive potential of online tools stands out as a positive outcome in this study.

It is worth noting that negative themes appeared more frequently than positive ones in the qualitative findings. Several considerations may help explain why empowering experiences with social media were reported less often. First, participants were recruited from a clinical setting, where social media-native mothers may have been more likely to seek support due to perceived challenges or distress, which could have increased the prominence of negative experiences in their accounts. Second, within the Turkish sociocultural context, cultural values such as the tendency to avoid self-praise may lead mothers to understate personal achievements, while sharing difficulties and challenges more openly in supportive environments [84]. Third, negative experiences often carry stronger emotional intensity and are therefore more likely to be remembered and described in detail compared to positive ones [20]. This pattern should not be interpreted as an absence of positive effects, but rather as an indication of how mothers in the postpartum period tend to prioritize and frame their experiences.

The findings of this study highlight that social media can function both as a source of stress and as a supportive resource during the postpartum period, particularly for social media-native mothers. Negative effects are particularly pronounced when these mothers are exposed to unfiltered or unverified content, confronted with highly idealized portrayals of motherhood, or possess limited digital literacy skills. Conversely, positive outcomes tend to emerge when they have access to credible, expert-driven resources, critically evaluate the content they encounter, and benefit from a strong offline support network.

We strongly recommend incorporating a targeted “digital risk literacy” section into perinatal education programs, adding short social media overuse assessments to standard postpartum visits, and collaborating with software developers to design culturally specific mobile applications that provide breastfeeding advice, maternal mental health resources, and strategies for safe social media engagement. Such interventions may serve as an important preventive measure to protect maternal mental health and support informed decision-making in the postpartum period.

### Limitations

This study has several limitations. First, participants were recruited from a single hospital-based postpartum clinic in Türkiye, which may limit the generalizability of the findings. When interpreting the results, one should take into account local sociocultural factors such as strong breastfeeding norms, family involvement in infant care, and varied digital literacy. Second, potentially influential variables, including maternal mental health history, sleep quality, time devoted to infant care, and prior social media habits, were not assessed and may have influenced both anxiety levels and problematic social media behaviors. Third, the low and homogeneous SMDS-9 scores in the sample may have reduced the ability to detect significant associations. Fourth, mothers in the early postpartum period (first two weeks) were excluded to minimize the effects of physical recovery and hormonal changes; however, this may have underestimated the potential impact of problematic social media stressors. Finally, self-reported data may carry the risk of recall and social desirability bias. Future studies should employ multi-site sampling, assess additional variables, and adopt longitudinal designs, including the early postpartum period.

## 5. Conclusions

This study emphasizes that social media-native mothers, although not showing statistically significant associations between problematic social media use, breastfeeding intentions, and anxiety levels, may still represent a uniquely vulnerable group in the context of social media use. Younger mothers with higher education and better economic conditions were more prone to unhealthy engagement, while increasing maternal age was associated with reduced dependency on social media and higher breastfeeding rates. Approximately three-quarters of the participants demonstrated strong intentions to breastfeed.

Qualitative findings revealed mothers’ experiences of social comparison, guilt, and uncertainty in infant care, echoing prior studies showing that social media can simultaneously serve as a source of support and emotional strain. These insights suggest that problematic social media use may hold potential implications for maternal well-being and infant care, even if not evident in quantitative measures.

The findings highlight the need to design preventive interventions against problematic social media use, particularly for social media-native mothers and future generations. Possible solutions include the development of Social Media Risk Literacy training modules, clinical screening programs to assess social media overuse, and user-friendly mobile applications for maternal–infant health and breastfeeding. Integrating these components into perinatal education as a comprehensive *“Support Package”* could promote healthy online behaviors, strengthen breastfeeding practices, and improve maternal and infant outcomes. Addressing problematic social media use in modern motherhood is no longer optional but a necessity for protecting public health in the digital age.

## Figures and Tables

**Table 1:** Demographic information of the participants (N = 222).

Variables	Categories	*n* = 222	%
Education	Primary school	12	5.4
	Middle school	31	14.0
	High school	70	31.5
	University	109	49.1
Economic situation	Income is less than expenses	38	17.1
	Income equals expense	132	59.5
	Income is more than the expense	52	23.4
How many children do you have?	1	136	61.3
	2	63	28.4
	3	21	9.5
	4	2	0.9
Currently breastfeeding?	Yes	197	88.7
	No	25	11.3
How many times a day should you feed your baby?	1–3	6	3.2
	Between 3–7	5	2.3
	Between 7–10	41	18.5
	More than 10	145	65.3
	Not breastfeeding	25	10.8
If not breastfeeding, why did you stop breastfeeding?	Pregnancy	1	5.88
	Breast rejection	1	5.88
	Health	13	76.47
	Curdling of milk	2	11.76
Switching to supplementary food	Yes	44	19.8
	No	178	80.2
Age	Mean ± SD	23.95 ± 2.86	
	Minimum–maximum	18–34	
How many weeks old is the baby?	Mean ± SD	5.39 ± 2.73	
	Minimum–maximum	2–24	
Duration of breastfeeding for the previous baby (Month).	Mean ± SD	17.15 ± 8.36	
	Minimum–maximum	0–24	
At what age did you switch to supplementary food?	Mean ± SD	3.75 ± 2.01	
	Minimum–maximum	1–6	

**Table 2:** Descriptive statistics of the scales (STAI-T, IIFAS, SMDS-9, IFI) used in the study.

Scales	Mean ± SD	Min–Max	Distortion (Skewness)	Kurtosis (Kurtosis)	Questions of Scales	Cronbach Alpha
1—The State-Trait Anxiety Inventory (STAI-T)	42.89 ± 8.41	21–70	0.178	0.048	20	0.826
2—The Iowa Infant Feeding Attitude Scale (IIFAS)	66.72 ± 8.09	30–84	−0.864	1880	17	0.665
3—The Social Media Disorder Scale (SMDS-9)	8.70 ± 6.10	0–32	0.782	0.544	9	0.799
4—Infant Feeding Intentions (IFI) Scale	Categories	n	%		5	0.838
	Low	6	2.7			
	Middle	49	22.1			
	Strong	167	75.2			

**Table 3:** Relations between participants’ scale scores (STAI-T, IIFAS, SMDS-9).

Scales	*r & p*	Total Score STAI-T	Total Score IIFAS	Total Score SMDS-9
1—The State-Trait Anxiety Inventory (STAI-T)	*r* *p*	1		
2—The Iowa Infant Feeding Attitude Scale (IIFAS)	*r* *p*	−0.126 0.062	1	
3—The Social Media Disorder Scale (SMDS-9)	*r* *p*	0.076 0.260	−0.110 0.103	1

**Table 4:** Comparison of scale scores (STAI-T, IIFAS, SMDS-9) corresponding to the IFI levels (N = 222).

Scales	IFI Levels	*n*	Average ± SD	F Value	*p*
1—The State-Trait Anxiety Inventory (STAI-T)	Low	6	42.33 ± 10.11	2143	0.120
	Middle	49	45.08 ± 9.38		
	Strong	167	42.27 ± 7.99		
2—The Iowa Infant Feeding Attitude Scale (IIFAS)	Low	6	58 ± 10.58	6552	0.002
	Middle	49	64.63 ± 8.27		
	Strong	167	67.65 ± 7.68		
3—The Social Media Disorder Scale (SMDS-9)	Low	6	10.16 ± 3.76	0.590	0.555
	Middle	49	9.36 ± 6.34		
	Strong	167	8.46 ± 6.11		

One-way ANOVA.

**Table 5:** Comparison of scale scores according to some demographic characteristics of the participants (N = 222).

Scales	Education Status Levels	*n*	Average ± SD	F Value	*p*
1—The State-Trait Anxiety Inventory (STAI-T) Score	Primary school	12	44.5 ± 8.28	0.163	0.921
	Middle school	31	43.06 ± 8.64		
	High school	70	42.75 ± 8.54		
	University	109	42.76 ± 8.37		
2—The Iowa Infant Feeding Attitude Scale (IIFAS) Score	Primary school	12	63.75 ± 8.01	0.600	0.616
	Middle school	31	66.64 ± 8.04		
	High school	70	66.77 ± 7.04		
	University	109	67.05 ± 8.75		
3—The Social Media Disorder Scale (SMDS-9) Score	Primary school	12	7.5 ± 6.34	2658	0.049
	Middle school	31	6.54 ± 5.04		
	High school	70	10.05 ± 6.12		
	University	109	8.58 ± 6.20		

**Table 6:** Comparison of scale scores according to participants’ income level (N = 222).

Scales	Income Levels	*n*	Average ± SD	F Value	*p*
1—The State-Trait Anxiety Inventory (STAI-T) Score	Income is less than expenses	38	43.68 ± 8.81	1532	0.208
	Income equals expense	132	43.37 ± 8.38		
	Income is more than the expense	52	41.09 ± 8.08		
2—The Iowa Infant Feeding Attitude Scale (IIFAS) Score	Income is less than expenses	38	67.13 ± 10.66	0.117	0.889
	Income equals expense	132	66.51 ± 7.81		
	Income is more than the expense	52	66.98 ± 6.62		
3—The Social Media Disorder Scale (SMDS-9) Score	Income is less than expenses	38	6.68 ± 5.54	3163	0.044
	Income equals expense	132	8.81 ± 5.94		
	Income is more than the expense	52	9.90 ± 6.63		

**Table 7:** Comparison of some characteristics of the baby and maternal age variables with the scales score.

Variables		The State-Trait Anxiety Inventory (STAI-T) Score	The Iowa Infant Feeding Attitude Scale (IIFAS) Score	The Social Media Disorder Scale (SMDS-9) Score
Age	*r*	0.043	0.147	−0.231
	*p*	0.525	0.028	0.001
How many weeks old is the baby?	*r*	0.061	0.114	0.044
	*p*	0.368	0.090	0.515
Duration of breastfeeding for the previous baby.	*r*	−0.242	0.090	0.149
	*p*	0.027	0.414	0.177
At what age does a baby start to eat supplementary food?	*r*	−0.018	0.508	−0.245
	*p*	0.932	0.008	0.227

**Table 8:** The statistics between the demographic characteristics and the baby feeding intention scale scores (N = 222).

Scales	Categories	IFI Levels			Chi Square	*p*
		Low *n* (%)	Middle *n* (%)	Strong *n* (%)		
Education Status	Primary school	1 (8.3)	2 (16.7)	9 (75)	0.076	0.823
	Middle school	1 (3.2)	4 (12.9)	26 (83.9)		
	High school	3 (4.3)	18 (25.7)	49 (70)		
	University	1 (0.9)	25 (22.9)	83 (76.1)		
Economic situation	Income is less than expenses	0 (0)	7 (18.4)	31 (81.6)	3694	0.059
	Income equals expense	3 (2.3)	28 (21.2)	101 (76.5)		
	Income is more than the expense	3 (5.8)	14 (26.9)	35 (67.3)		
The current number of children.	1	3 (2.2)	34 (25)	99 (72.8)	0.727	0.450
	2	2(3.2)	12 (19)	49 (77.8)		
	3	1 (4.8)	3 (14.2)	17 (81)		
	4	0 (0)	0 (0)	2 (100)		
Is the mother breastfeeding the baby?	Yes	2 (1)	43(21.8)	152 (77.2)	9007	0.005
	No	4 (16)	6 (24)	15 (60)		
How many times a day does she breastfeed?	1–3	1 (33.3)	2 (4.7)	4 (2.6)	5873	0.017
	Between 3–7	1 (33.3)	1 (2.3)	3 (60)		
	Between 7–10	1 (33.3)	9 (20.9)	31 (2)		
	More than 10	0 (0)	31 (72.1)	114 (75)		
Switching to supplementary food	Yes	4 (66.7)	12 (24.5)	28 (16.8)	6969	0.012
	No	2 (33.3)	37 (75.5)	139 (83.2)		

**Table 9:** Comparison of IFI scores according to selected infant characteristics and maternal age variables. (N = 222).

Scales	IFI Levels	*n*	Average ± SD	F Value	*p*
Age	Low	6	23.83 ± 2.99	0.078	0.925
	Middle	49	23.81 ± 2.88		
	Strong	167	23.99 ± 2.86		
How many weeks old is the baby?	Low	6	5.08 ± 2.24	0.039	0.961
	Middle	49	5.40 ± 2.76		
	Strong	167	5.40 ± 2.75		
Duration of breastfeeding the previous baby	Low	6	4 ± 6.92	4931	0.010
	Middle	49	15.50 ± 8.95		
	Strong	167	18.17 ± 7.78		
At what age does a baby start to eat supplementary food?	Low	6	5	0.193	0.826
	Middle	49	3.75 ± 1.90		
	Strong	167	3.67 ± 2.18		

## Data Availability

The raw data supporting the findings of this study are not publicly available due to ethical concerns and the inclusion of sensitive participant information. However, the data can be made available from the corresponding author upon reasonable request, in accordance with institutional and ethical guidelines.
